# C-terminal part of alpha-synuclein mediates its activity in promoting proliferation of dopaminergic cells

**DOI:** 10.1186/1750-1326-7-S1-S21

**Published:** 2012-02-07

**Authors:** Juanjuan Yin, Tao Wang, Junyan Han, Chen Zhang, Qiulan Ma, Xin Li, Furong Cheng, Guangwei Liu, Yaohua Li, Kenji Uéda, Piu Chan, Shun Yu

**Affiliations:** 1 Department of Neurobiology and Sino-Japan Joint Laboratory for Neurodegenerative Diseases, Beijing Institute of Geriatrics, Xuanwu Hospital of China Capital Medical University, Beijing 100053, China; 2 Institute for Hypoxia Medicine, Xuanwu Hospital of China Capital Medical University, Beijing 100053, China; 3 Division of Psychobiology, Tokyo Institute of Psychiatry, Tokyo 156-8585, Japan

## Background

Although abnormal aggregation of alpha-synuclein (α-syn) is involved in several neurodegenerative diseases, its biological functions remain poorly understood, which limits our understanding of its pathogenic mechanisms. α-Syn has been shown to exhibit a MAP-like activity and promote the assembly of microtubules. Since microtubules play a pivotal role in proliferative cell division, it is possible that α-syn affects cell proliferation by facilitating microtubule assembly. The role of α-syn in promoting cell proliferation was reported previously in PC12 dopaminergic cells overexpressing α-syn. Here, we extended this study aiming at finding the association between the cell proliferation effect of α-syn and its microtubule assembly activity, and identifying the potential active domain for the effect α-syn on cell proliferation.

## Method

By exploiting the property that the 11-mer repeats of synuclein molecules are able to mediate a rapid intracellular translocation of these proteins across the plasma membrane without being degraded by the cellular proteolytic system, we added recombinant full-length α-syn (wild type and A53T and A30P mutants) and β-syn to the culture medium of MES23.5 dopaminergic cells, and observed their intracellular translocation, subcellular distribution and effects on cell proliferation.

## Result

We found that all the synuclein molecules could enter the cells where they were localized in both the cytoplasm and nucleus. However, only the wild-type α-syn, which had been shown to have microtubule assembly activity, was able to promote proliferation of the MES23.5 cells. The A53T and A30P mutant α-syn as well as β-syn, which had been proved not to possess microtubule assembly activity, did not exhibit any effect on cell proliferation. Since the α-syn activity in microtubule assembly was shown to be related to its specific functional domain, we then generated different functional fragments (N-terminal 1-65aa, NAC 66-95aa and C-terminal 96-140aa) and tested their activities in cell proliferation. We showed that all the α-syn fragments could enter the cells, but with different subcellular localizations. The N-terminal and NAC fragments were localized in the cytoplasm and the C-terminal fragment mainly in the nucleus. In accordance with the activity for the C-terminal part of α-syn in microtubule assembly, only the NAC and C-terminal fragments exhibited the activity in cell proliferation. The N-terminal fragment without microtubule assembly activity did not promote cell proliferation.

## Conclusion

The above results suggest that the α-syn function in promoting cell proliferation is associated with its microtubule assembly activity with the functional domain localized in its C-terminal part.

